# An innovative system for 3D clinical photography in the resource-limited settings

**DOI:** 10.1186/1479-5876-12-169

**Published:** 2014-06-15

**Authors:** Saharnaz Baghdadchi, Kimberly Liu, Jacquelyn Knapp, Gabriel Prager, Susannah Graves, Kevan Akrami, Rolanda Manuel, Rui Bastos, Erin Reid, Dennis Carson, Sadik Esener, Joseph Carson, Yu-Tsueng Liu

**Affiliations:** 1 University of California San Diego, La Jolla, CA, California; 2 California Institute of Technology, Pasadena, CA, USA; 3 Jefferson Medical College, Philadelphia, PA, USA; 4 University of California San Francisco, San Francisco, CA, USA; 5 University of Eduardo Mondlane, Maputo, Mozambique; 6 College of Charleston, Charleston, SC, USA

## Abstract

**Background:**

Kaposi’s sarcoma (KS) is the most frequently occurring cancer in Mozambique among men and the second most frequently occurring cancer among women. Effective therapeutic treatments for KS are poorly understood in this area. There is an unmet need to develop a simple but accurate tool for improved monitoring and diagnosis in a resource-limited setting. Standardized clinical photographs have been considered to be an essential part of the evaluation.

**Methods:**

When a therapeutic response is achieved, nodular KS often exhibits a reduction of the thickness without a change in the base area of the lesion. To evaluate the vertical space along with other characters of a KS lesion, we have created an innovative imaging system with a consumer light-field camera attached to a miniature “photography studio” adaptor. The image file can be further processed by computational methods for quantification.

**Results:**

With this novel imaging system, each high-quality 3D image was consistently obtained with a single camera shot at bedside by minimally trained personnel. After computational processing, all-focused photos and measurable 3D parameters were obtained. More than 80 KS image sets were processed in a semi-automated fashion.

**Conclusions:**

In this proof-of-concept study, the feasibility to use a simple, low-cost and user-friendly system has been established for future clinical study to monitor KS therapeutic response. This 3D imaging system can be also applied to obtain standardized clinical photographs for other diseases.

## Background

It cannot be over-emphasized that high-quality images are critical for clinical decisions. The current trend of establishing electronic health record (EHR) in the resource-rich settings and mobile device-based health care system to reach patients in the resource-limited settings makes standardization and acquisition of high-quality clinical photography an imperative issue. Standardization of clinical images is not only crucial for disease diagnosis, patient follow-up, clinical communication and medical education, but also for computer-aided clinical management. While nearly all of the radiological imaging systems, e.g. plain X ray, CT, MRI and ultrasound, have standard guidelines for obtaining and interpreting images, there is little consensus or effort in clinical photography except for a few areas, such as presenting the outcome of a plastic surgery. In fact, many diseases can be diagnosed by their characteristic presentations before any further confirmation tests. Several medical journals, such as the New England Journal of Medicine (Images in Clinical Medicine and Image Challenge) [1], Journal of American Medical Association (Clinical Challenge) [2], British Medical Journal (Picture Quiz), etc., frequently publish typical diseases that may be diagnosed through the analysis of a single clear and informative image.

In 1989, The AIDS Clinical Trial Group (ACTG) proposed a KS staging system using a three-tiered system that classified the extent of tumor (T) involvement, the status of a patient’s immune system (I) assessed by CD4 cell count, and the severity of their systemic illness (S) as “good risk” or “poor risk” [3]. The ACTG staging system and variants that are used to assess KS therapeutic response may include lesion counts, assessment of lesion color, nodularity, ulceration and associated edema, measurement of sum of the product of the diameters of 5 marker lesions, and evaluation for oral KS and secondary effects of KS [3-6]. The color and degree of nodularity of KS lesions are difficult to quantify, but often provide important information about response to treatment [3]. While conventional photography is probably the single most useful technique for imaging KS, it does not provide enough information about tumor nodularity and other important characteristics.

In contrast to what many would think, obtaining high-quality clinical photography is not trivial [7-9]. The suggestion of using professional clinical photographers in the professional studio settings to control the surrounding environment for light, angle etc. is not overstated for producing clear and informative photographs. However, a photographer cannot focus on a relevant lesion unless he or she has been told by an experienced clinician. Therefore, it would be difficult to generate informative images without knowing where to focus. To overcome this dilemma and to ease the technical hurdles, we have developed an innovative approach by taking advantage of the light-field photography in conjunction with a design of a miniaturized “photography studio” to control environment and standardize the image presentation. Here we present a proof-of-concept approach for developing a 3D image based system to monitor therapeutic response of Kaposi’s sarcoma in Mozambique. While this system is developed for clinical photography, applications exist for other areas that require collecting evidence by non-professional personnel in field, including applications for veterinary, agriculture, and even crime investigation.

## Methods

### 3D imaging system

#### Camera

**Figure 1:**
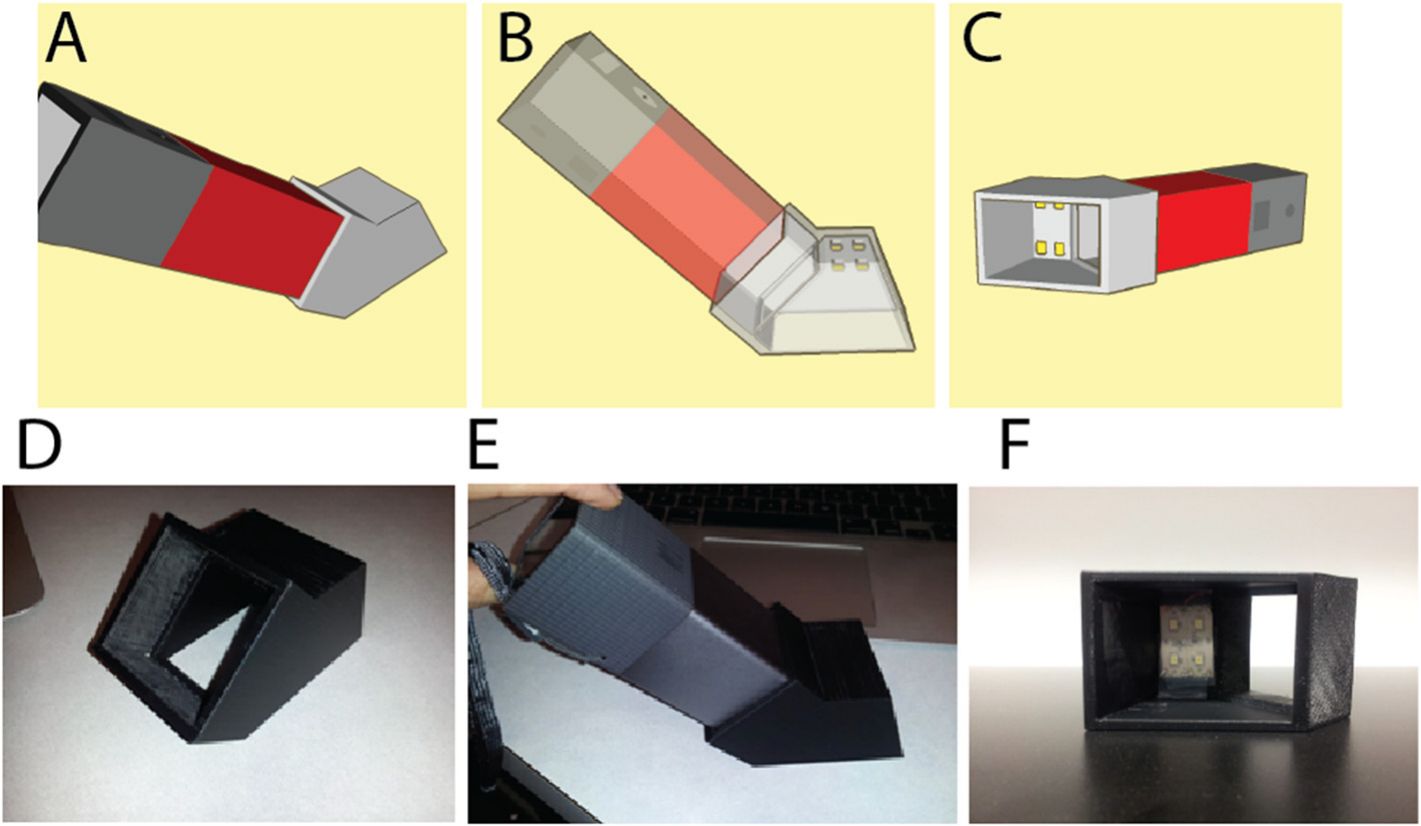
**Design and Assembly of the miniature photography studio adaptor**. The adapter was designed with SolidWorks CAD program **(A-C)** and was 3D printed **(D)**. The adapter can be attached to a Lytro camera at 45° angle **(E)** and provides illumination through LEDs **(F)**.

A consumer light field camera, Lytro [10-13], was used to develop a simple and reliable imaging system for producing 3D clinical photographs. This camera has an unconventional physical appearance that is convenient for our imaging system design. It has a rectangular prism shape (1.61x1.61x4.41 inches, 7.6 ounces) with the lens at one end and a touch screen at the other (Figure 1E). Instead of focusing on a single focal plane, Lytro, a digital light field camera, samples each individual ray of light that contributes to an image [10]. To record the light field inside the Lytro camera, a microlens array is placed in front of the photosensor. This idea originated from an earlier “plenoptic camera” proposed by Adelson et al. [14]. In the Lytro camera, each microlens covers a small array of photosensor pixels. The microlens separates the light that strikes it, and focuses it into a tiny image on this array, forming a miniature picture of the incoming light. The raw image is not readily recognizable by human eyes. Through ray-tracing techniques, images of multiple focal planes can be generated.

#### Miniature “Photography Studio” Adaptor

The adaptor was designed to create a standardized background environment for the Lytro snapshot by fulfilling the mechanical, optical and post processing requirements. It is to be placed at the front end of the Lytro camera and holds the camera at 45 degree with respect to the skin (Figure 1). The SolidWorks software was used to design the mechanical structure of the adaptor. It consists of two conceptual parts that are attached together and then 3D printed as a single element. The Dimension 1200es 3D Printer from Stratasys Company was used for this purpose. The layer thickness of the printer is 0.254 mm (.010 in.) and it uses ABSplus (acrylonitrile butadiene styrene) [15] that is resistant to alcohol and is therefore suitable for sterilization purposes. Different views of the CAD (Computer-aided design) structure can be seen in Figure 1A-1C. Designed to enable optimal object illumination, the adaptor consists of a matrix of two by two strip LEDs (Figure 1C and 1F) along with a switching circuit that are installed and fitted on the adaptor’s top surface.

### Kaposi sarcoma lesions

Anonymous clinical photographs of KS lesions were obtained from the Dermatology Service at the Maputo Central Hospital (MCH) in Maputo, Mozambique. The great majority of patients (>90%) with KS at MCH are infected with HIV, and many are diagnosed as HIV positive at the time that they present with KS. Ten to fifteen patients with KS are admitted to the Dermatology service at MCH per month. Currently, MCH physicians document treatment response in the clinical chart using sketch diagrams and verbal descriptions.

### All-focused image and 3D image model

Using a program modified from an open source python library, lfp-reader-2.0.0 [16], the original Lytro image file (.lfp) was extracted to obtain JPEG images (usually 12) of different focused depths, and a corresponding depth map was generated. We used batch processing to generate 12 JPEG files, a depth map and an all-focused image. The procedure was controlled through a graphic user interface (GUI) for ease of code execution. The final data was outputted into individual folders for each Lytro image set.

Another GUI program was created for rendering the 3D model of each Lytro image data using the aforementioned depth map and all-focused image. This program is a modification of the code from a command-line based C program [17].

We also used a computational software program, Mathematica, to perform a final 3D display with interactive rotation capabilities. As inputs, Mathematica receives the depth map, stored in ASCII format, as well as the corresponding all-focused JPEG. Our customized display script calls the Mathematica function ListSurfacePlot3D to generate the 3D shape from the depth map. Within this function, Mathematica’s PlotStyle option is called to texture the rendered 3D shape with the all-focused JPEG. Once the fully textured 3D data is displayed, Mathematica’s standard interactive tools may be used to rotate the object around any of the three dimensional axes.

### Evaluation of relative volume change

The relative volume change was evaluated by a prototype software program that uses the projected area of a lesion to derive an average radius. The average radius is then used to approximate a three-dimensional volume. The prototype software program carries out these procedures by first counting the number of pixels, in a single 2D digital image, occupied by the object. This number of pixels represents the object’s projected area, in relative units. In order to convert this into a signature radius, the program generates a hypothetical circle whose area, in pixel units, is equal to the object’s projected area. Using the formula for the area of a circle, *Area = π(radius)*^*2*^, the program solves for a radius, in units of pixels, using the aforementioned measured area. This procedure is repeated for each unique perspective snapshot, and the radii are averaged. The camera adaptor (Figure 1) ensures that the distance to the object remains constant between the unique perspective snapshots. The averaged radius value is then converted into a volume, in units of cubic pixels, using the formula for a sphere, *Volume = 4/3 π(radius)*^*3*^. This value represents a signature relative volume, which may be used to measure fractional relative changes between a lesion that is photographed at different points in time.

A challenge for the aforementioned technique is accurately identifying, in the digital image, the visual boundary between the lesion and the background skin surface, a step that is necessary in order for the program to accurately calculate the projected area. In our current proof-of-concept program, we have successfully applied two different methods for determining this boundary: (1) by utilizing the information in the depth map to separate the lesion from the background surface through differences in depth; and (2) by visual inspection and manual input from a user who traces out the edge of the lesion on a digital touchscreen. In test cases, both strategies proved effective for a range of images, though each method has its strengths and limitations. Method (1) successfully works as long as program parameters are carefully optimized for typical depth map shapes. While proving effective for a range of lesion types, it can be a risky strategy for lesions that may represent diverse sizes, depths, and viewing angles. For example, it has particular difficulties for shallow lesions, where there is little change in height between the lesion and the skin. Despite these drawbacks, an advantage of this method is that it is fully automated, once parameters are well tuned, and therefore avoids potential errors associated with human error. Method (2), lesion boundary identification by visual inspection, has proven in our tests to be the most accurate method and the most capable method for accommodating diverse lesion sizes and shapes. Its primary disadvantage is that it requires human input and therefore adds an element of potential subjectivity, and potential human error, into the procedure.

## Results

### Miniature “Photography Studio” Adaptor

The adaptor is used to maintain constant geometric conditions of the environment and to provide controllable illumination. Its development was motivated by our having experienced initial difficulty to obtain a reliable 3D image using the Lytro camera alone. The adaptor simplifies the effort to generate a 3D image with a single shot and to compare the images of lesions before and after treatment.

### All-focused image and 3D image model

**Figure 2:**
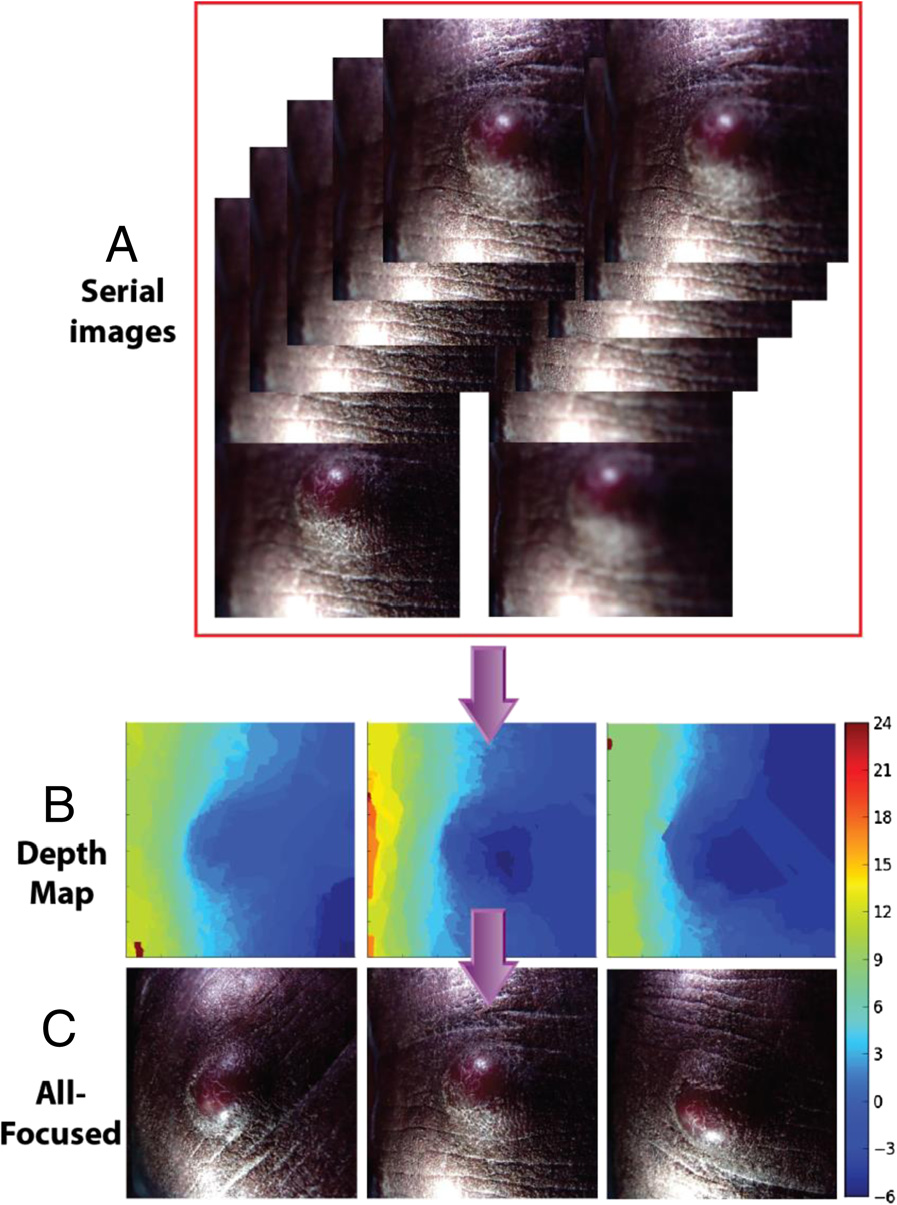
**Construction of all-focused images**. The original Lytro files were computationally extracted to output a series of images with a range of focus settings **(A)** and corresponding depths for each position in the image (**B**, depth map). Even with just a single Lytro snapshot (which contains 12 frames), an all-focused **(C)** image and a 3D model (Figure 3) can be constructed by combining the information from A and B. There are 3 all-focused images on panel C. They are derived from 3 single Lytro shots at different horizontal angles of the same KS lesion (<1 cm). Only one set of the serial images are shown (for the picture on the middle) in panel A.

The original Lytro files (.lfp) were computationally extracted to output a series of images with a range of focus settings (Figure 2A) and to output a corresponding depth for each position in the image (B, depth map) (Figure 2). We have consistently obtained 12 JPEG files from each file extraction after we used the adapter described earlier. Before our current system was set up, the results were inconsistent, even after we chose a well lit space to improve illumination and used a tripod to control spatial factors (angle, distance etc.). We have observed that the effective depth of a Lytro snapshot can be severely compromised when there are background objects at far distance. The simple, miniature, “photography studio” adaptor substantially resolved previous issues of inconsistent image quality.

**Figure 3:**
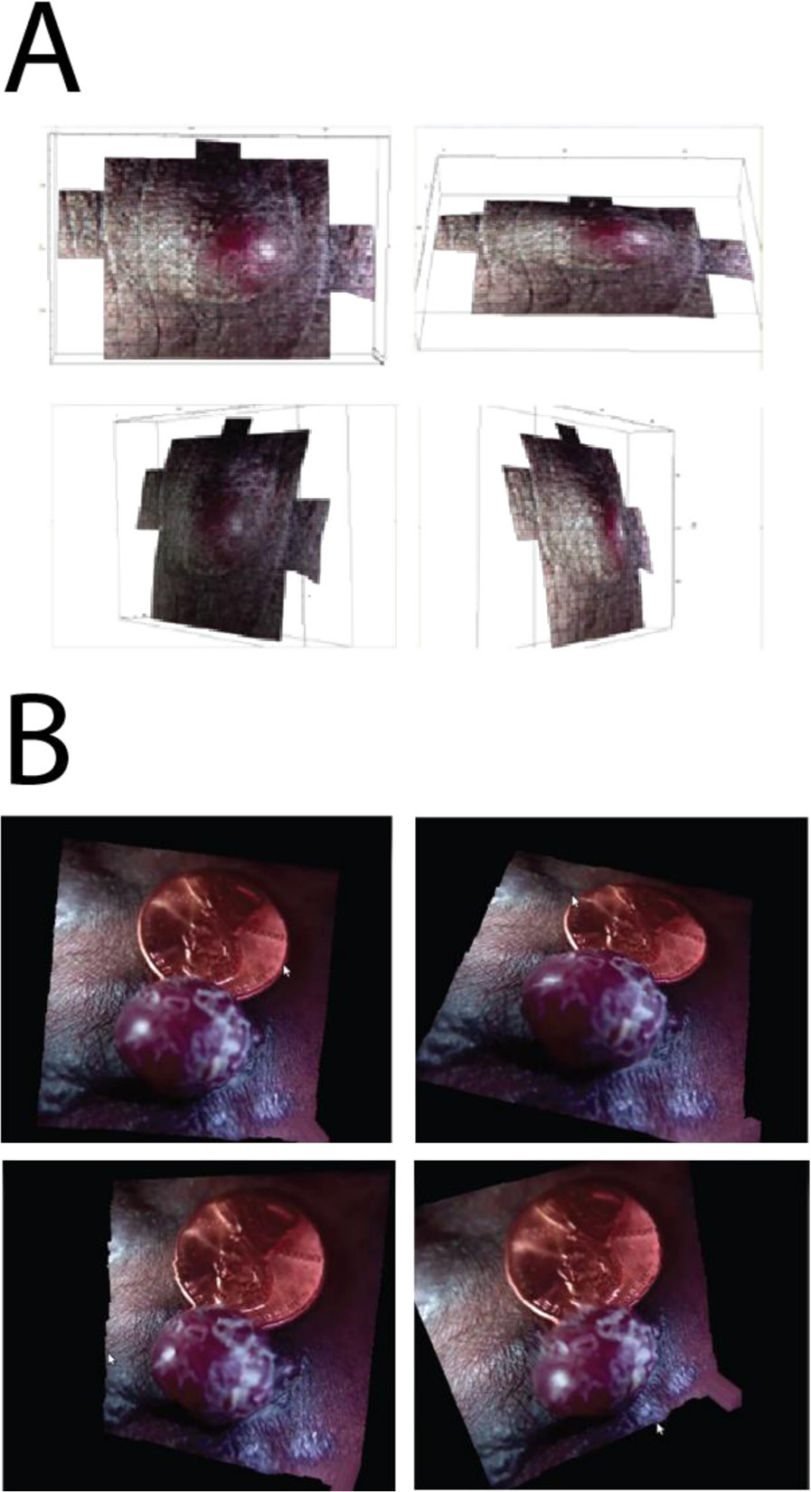
**A 3D rendering of Kaposi’s Sarcoma, generated from a single Lytro snapshot processed by a prototype software using (A) commercial and (B) open-source platforms**. Two examples are shown (Supplementary video included, Additional file 1: Video S1 and Additional file 2: Video S2).

The concept and operation of creating the all-focused image (Figure 2) and 3D model (Figure 3) are similar. Both procedures take advantage of the image files at different focal planes and and also the depth map, but separate computational programs are applied to generate two different outputs. For clinical application, the all-focused image can be used for quick screening purposes as the size of the file is small, usually around 80 kb (30–220 kb based on 185 snapshots). The size is appropriate as an attachment through cell phone transmission. On the other hand, the 3D image model provides a more realistic view of a lesion, but is more computationally intensive. To simplify the operation, we created a custom GUI program to facilitate users with little or no training in knowing how to process image files. This is particularly important in the resource-limited settings. In addition, we use both commercial (Figure 3A, Additional file 1: Video S1) and open source (Figure 3B, Additional file 2: Video S2) software programs to meet various needs (see Methods section).The scheme in Figure 2 also reveals the simplicity of this system, in terms of the level of human involvement, despite very complex computational operations. The figure represents 3 Lytro snapshots, which result in 36 (12 × 3) JPEG images of different focal planes, with multiple angles (0, 120, and 240 degrees, horizontally from a reference point). Therefore, using our system, 36 sub-spaces were sampled close to the lesion with very little human effort.

### Evaluation of relative volume change

**Figure 4:**
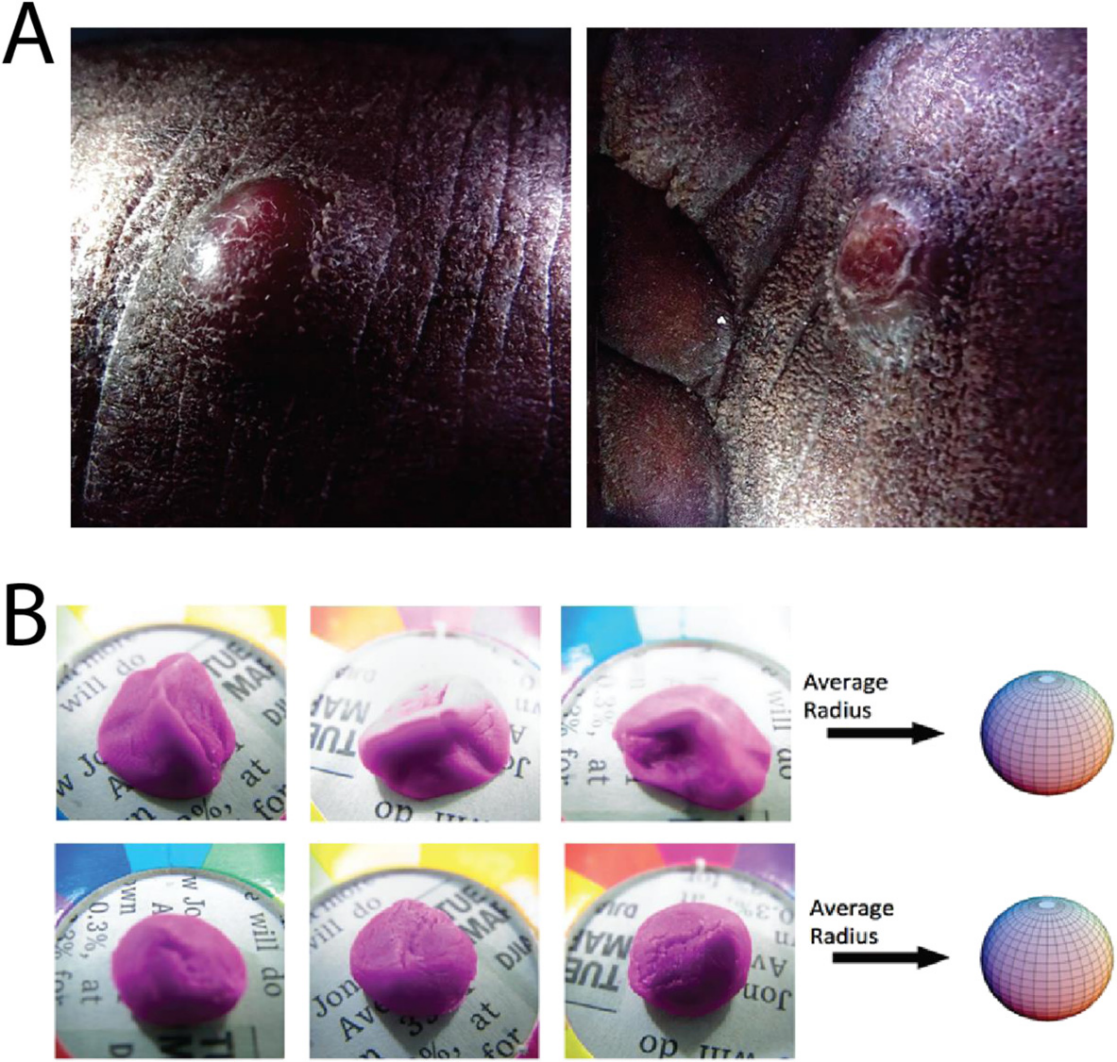
**Changes of nodular KS lesions. (A)** A pair of nodular KS lesions to demonstrate before (left) and after (right) treatment over 2 weeks. **(B)** The relative volume of two clay samples (one on top row and one on bottom row) is measured using the prototype image analysis software. Each column represents one of three independent angle perspectives. The program uses the projected 2D shape to measure an average radius. This radius is then used to determine a volume. For measuring relative changes in volume, the program can accurately deliver results with an uncertainty of 4%, as long as the changes represent solely an enlargement or diminution in an original shape, as opposed to a complete change in shape. In instances where the overall shape changes completely (for instance, like from a long cigar shape to a half sphere), changes in relative volume may be measured with an uncertainty of about 34%.

As previously described, a useful tool to monitor therapeutic response of KS is to evaluate changes of nodular lesions by observing the lesion’s thickness and volume over time [18]. However, the follow-up period for each patient was very short (<2 weeks) in our proof-of-concept study (see an example in Figure 4A). Therefore, we chose to use a model clay system (Figure 4B) to demonstrate a simple measurement that may be applied in a more comprehensive clinical study. Notably, clay molding allows a more precise measurement of the correlation between the true volume and the volume derived by computational modeling.While a full 3D rendering provides the most powerful information, a measurement of a lesion’s volume can also be achieved using conventional 2D snapshots (taken with either a traditional digital camera or a Lytro camera) and applying a software algorithm to approximate true volume from the projected 2D shape. Multiple perspective snapshots are not absolutely required, but the overall uncertainty improves with each independent perspective. The prototype software program, written by our group, measures the projected area of a lesion and uses that information to derive an average radius. This average radius may then be used to approximate a three-dimensional volume. Figure 4B demonstrates the effectiveness of the software prototype when applied to a set of digital images of randomly shaped clay models. To evaluate the uncertainty on relative volume, we measured differences between observed relative volume, as measured by the prototype program, and true relative volume, as determined by independent knowledge of the clay source. In order to include a diverse range of potential shapes in our uncertainty evaluation, we supplemented these test case images with simulated images of a wide variety of target shapes, ranging from that of symmetrical sphere-like objects to long flattened shapes with extreme differences in relative dimensions (like 10:1 ratio between height and width). After processing both true and simulated images through the prototype program, the results of these tests indicated that, when three snapshots of a target are taken from independent perspective angles, the technique can successfully measure relative changes in volume with an uncertainty of 4%, as long as the second epoch shape represents an approximately enlarged, contracted, or unchanged version of the first epoch shape; this scenario represents the vast majority of KS diagnoses. In instances where the overall shape changes completely (for instance, like from a long cigar shape to a half sphere), changes in relative volume may be measured with an uncertainty of about 34%. The information gathered from this approach may be combined with that from other diagnosis techniques in order to flag growths that may deserve further evaluation by a clinician.

## Discussion

An increasing number of digital technologies are being applied to promote virtual communication between patients and healthcare providers and medical specialists, without requiring face-to-face encounters. Accordingly, there has been a surging interest in utilizing mobile technology to meet the rising healthcare demand in developed countries, as well as to serve people living in a more resource-limited world.

An opportunity exists to apply a novel digital imaging technology to improve diagnosis and long-term monitoring of Kaposi’s Sarcoma (KS) in a resource limited setting. A recent report from Mozambique has shown that KS is the most frequent cancer among men and the second most frequent among women [19]. Remarkably, 20% (296/1507) of cancers diagnosed at the Maputo Central Hospital (MCH) are KS. The therapeutic response of KS may be different between this region and western countries, providing some of the motivation for effective monitoring tools [20]. Furthermore, in many cases, multiple KS lesions develop simultaneously, and may progress and regress independently. Unfortunately, relatively little is known about the varying response to treatment, and about the most effective therapeutic regimens [21]. Infrastructural inadequacies in the provisions of cancer care in sub-Saharan Africa pose great challenges for precise therapeutic monitoring. Therefore, there is an unmet need to develop a simple, but accurate, tool for improving diagnosis and long-term monitoring in such a resource-limited setting. While color and thickness of KS lesions are difficult to quantify, they provide important information about response to treatment [3]. Our simple and inexpensive imaging system and analytic software will resolve such a dilemma. Furthermore, the single processed all-focused images of the KS lesions were mostly around 80 kb (30–220 kb for 185 snapshots). This file size is transmissible through a mobile phone for preliminary diagnosis or triage.

The myriad benefits of such a tool should not be underestimated. In medicine, as in many fields, a picture is often worth a thousand words. Photographs are important tools for recording and tracking the progression of visible lesions. Effective utilization of photographic techniques can help reduce the patient and physician’s anxiety about adequate detection of lesion changes, help early identification and treatment of malignancy, and lead to fewer invasive procedures [22]. However, acquiring a clear, informative photo is not trivial. Apparently, one of the most obvious problems with conventional photography is the burden of accurate focus on the target. Our recent adoption of an emerging technology, light field photography using the Lytro camera, succeeds in capturing multi-focal images with a single photo shot. However, we and others have often experienced frustrations in acquiring such ideal images [13].Combining theoretical and experimental approaches, we were able to design an adaptor to serve as a miniature “photography studio” that enables reliable and predictable 3D image acquisition (Figure 1). Such an adaptor provides a controllable light source and enables standardized images (with a fixed camera-to-object distance and angle) for automated computational analysis. This is a very important step since, without this adaptor, image quality can suffer from day-to-day and person-to-person variations. Moreover, it makes the operation simple and intuitive, as one only needs the same level of skill required to use a smartphone or a digital camera. While our current software system is capable of producing a 3D image from a single Lytro snapshot (see Figures 2 and 3), our next-generation software should also be able to take advantage of instances where the clinical staff collects multiple snapshots to provide more comprehensive data.We envision the light field camera as a useful general tool for clinical photography. There is no need to select focal planes because the Lytro camera records all of the light rays within a distance based on the setting. This is a very attractive function for a busy clinic that may lack trained personnel to take medical photography. Importantly, a clinician can review the images at other focal planes that may not be foreseen as necessary, but become crucial when a disease progresses. Furthermore, an all-focused image (Figure 2) or a 3D rotatable model can be created computationally, as shown in Figure 3. While we used a commercially available program (e.g. Mathematica) for fast, prototype-level, 3D image modeling, we plan to transition to an open-source, 3D modeling program. This final version is intended to integrate and streamline every aspect of the image processing, enabling a user-friendly automatic system that can be used in the resource limited settings.

For surface volumetric analysis, we used a simple prototype software program, as previously described. There are many other computational methods for 3D volume measurement [23]. Clinically, it has been discussed whether more sophisticated medical imaging methods, such as CT and MRI, are more useful for measuring clinical outcomes [24]. It appears that the usefulness of a method is not only associated with the power of the computational algorithm, but also the limitation of the available technologies and practical requirements of standardization. Historically, the World Health Organization (WHO) proposed, more than 30 years ago, a system for obtaining cross-sectional images as a biomarker for therapeutic response [25]. For example, in this system a partial response is designated when bi-dimensional measurement of a single lesion shows greater than 50% reduction in cross-sectional area (as measured by perpendicular diameters). While the WHO criteria remain useful, an attempt to further simplify and standardize methods has been recently made [26,27]. The response evaluation criteria in solid tumors (RECIST) were proposed by the European Organization for Research and Treatment of Cancer (EORTC) in collaboration with the National Cancer Institute (NCI) and the National Cancer Institute of Canada Clinical Trials Group. Interestingly, the RECIST criteria only made use of the one-dimensional measurement rather than the product of two perpendicular linear measurements. According to their system, a partial response is defined as at least a 30% decrease in the sum of the longest diameter of the target lesions. It appears that the RECIST criteria were designed to simplify the way to gather and record information, not necessarily to increase accuracy. Whether 1D, 2D or 3D models are more useful seems to be associated with the nature of lesions, and will be constantly debated [24,27-29]. Nevertheless, the variations of measurement should be reasonably small if the method is to be useful. In our study (Figure 4), the simple technique can successfully measure relative changes in volume (for longitudinal follow-up) with an uncertainty of 4%, which is useful. But it would not be useful for horizontal comparison for two unrelated lesions (34% uncertainty). We are currently exploring different techniques and expect to use multiple methods depending on conditions.

As described earlier, a critical factor for volume estimation is identifying the boundary of a lesion. We have applied two non-mutual exclusive approaches. A third potential method, that we have yet to test, is boundary identification through changes in color. This strategy holds some promise as either an independent technique or as a supplement to the aforementioned methods. It holds the potential to be fully automatable, but may require optimization for typical patient skin color and typical lesion color characteristics. Ultimately, the optimal technique for lesion boundary identification is an area of investigation that requires further development, perhaps in conjunction with a careful analysis of typical lesion characteristics. It may require some combination of the three described techniques. We defer such a detailed investigation for future work.

Although our report focuses on the application of our imaging system to potentially monitor therapeutic response of KS, the same approach can be applied for screening skin lesions, especially for longitudinal follow-up [22]. While early diagnosis and treatment is the best strategy for reducing cancer mortality, overuse of expensive and/or invasive procedures increases morbidity and healthcare costs in developed countries [30,31] and is impractical in resource-limited settings. However, introduction of simple and inexpensive diagnostics and imaging devices has been proven to be useful in clinics. For example, the number-needed-to-excise (NNE) value, a measurement for accuracy of melanoma diagnosis, has been improved only in specialized and not in non-specialized clinical settings in a multicenter study over a 10-year period [32]. The main difference has been attributed to a larger use of diagnostic techniques, especially the simple and inexpensive dermatoscope, and digital monitoring [22,33]. As described earlier, our 3D imaging system may be especially useful in the telehelth settings as high-quality and relevant images can be obtained without extensive training of the operator. Moreover, it is possible to have a preliminary analysis by a software system as standardized images are obtained.

## Conclusions

In this proof-of-concept study, a feasibility to use a simple, low-cost and user-friendly system has been established for future clinical study to monitor KS therapeutic response. This innovative system can also be applied to obtain standardized clinical photographs for other diseases.

## Competing interests

The authors declare that they have no competing interests.

## Authors’ contributions

Conceived and designed the experiments: YL, JC, DC, SE; Performed the laboratory experiments: SB, KL, JC, YL; Performed the clinical work: JK, GP, SG, KA, RM, RB, ER; Analyzed the data: SB, KL, JC, YL; Wrote the paper: YL, JC, SB, KL. All authors read and approved the final manuscript.

## Supplementary Material

Additional file 1: Video S1 3D model display by Mathematica. Click here for file

Additional file 2: Video S2 3D model display by OpenGL. Click here for file
